# Editorial: Every cloud has a silver lining—COVID-19 and positive work outcomes

**DOI:** 10.3389/fpsyg.2023.1173250

**Published:** 2023-06-13

**Authors:** Kabiru Maitama Kura, Faridahwati Mohd Shamsudin, Kabiru Jinjiri Ringim, Mahmoud AlZgool

**Affiliations:** ^1^UTB School of Business, Universiti Teknologi Brunei, Gadong, Brunei; ^2^College of Business Administration, University of Sharjah, Sharjah, United Arab Emirates; ^3^ABU Business School, Ahmadu Bello University, Zaria, Nigeria; ^4^Department of Administrative and Financial Science, Gulf University, Sanad, Bahrain

**Keywords:** COVID-19, work outcomes, job stress, remote learning, work from home (WFH), organizational support, job satisfaction, innovative work behavior

We are extremely honored to be given the opportunity to guest edit this special edition of the Frontiers in Psychology Journal, which showcases outstanding articles related to the theme of the Research Topic. The call emphasizes that although many individuals and organizations tag the COVID-19 pandemic negatively, there are always constructive parts to it.

It was back in December 2019 when the world was told about the first case of a novel coronavirus disease (COVID-19) identified in China's Wuhan City in the Hubei Province (Sohrabi et al., 2020; Barai and Dhar, 2021). COVID-19 is a socio-economic disease that rapidly destroys nations' social and economic structures (Lupia et al., 2020; Shereen et al., 2020; Singh et al., 2020). On March 11, 2020, the Director-General of the World Health Organization (WHO) declared the COVID-19 outbreak a global pandemic (Ghebreyesus, 2020). Since then, the number of confirmed COVID-19 cases and deaths worldwide has increased daily. For example, the International Air Transport Association (IATA) estimated that the global airline industry lost over USD113 billion in passenger revenues to COVID-19 in 2020 (Pearce, 2020). The COVID-19 outbreak also prompted the closure of private businesses, governmental organizations, and other entities, resulting in lower employee productivity, decreased profitability, and higher fiscal deficit (Isabelle, 2020; Liang, 2020; Moody's Investors Service, 2020). Due to the magnitude of the economic and social consequences of COVID-19, researchers across various fields have carried out studies on this global pandemic.

The extant literature on COVID-19 has centered mainly on the effects of a pandemic on negative attitudinal and behavioral work outcomes (Hong et al., 2021; Reis et al., 2021). While such work is insightful, industrial and organizational psychologists also remark that the pandemic may play a crucial role in contributing to positive attitudinal and behavioral outcomes. However, as little is known about how the pandemic could drive workplace positivity, we invited the scientific communities for theoretical, empirical, and methodological contributions to refute the general belief that COVID-19 is entirely a negative phenomenon.

In response to our call, this Research Topic is pleased to share six accepted papers covering a wide range of topics on COVID-19 and positive work outcomes. The paper by Walugembe et al. reviews extant articles on adaptive behavior before and after the COVID-19 era in educational institutions. Specifically, the authors reviewed the outcomes of adaptive behavior and coping strategies over time. An interesting finding from their review is that although the COVID-19 pandemic has wreaked havoc on many aspects of life, educational institutions have benefited by transitioning to remote teaching and learning strategies.

Incorporating a two-round Delphi method, Tee et al. solicited expert opinions, judgment, and consensus to identify factors that positively impact higher education institutions (HEIs) by COVID-19. They identified seven factors: education reform, technology optimization, competency building and enhancement, student inclusivity, organization restructuring, work-life balance/humanities, and translational research. However, education reform was found to be the most crucial outcome of COVID-19 from the HEIs' perspective.

The paper by Čábelková et al. examines the role of emotional creativity (EC) in enhancing the adaptive, innovative behavior of university professors under the conditions of the COVID-19 epidemic. The results demonstrated that EC significantly improved the innovative adaptive behavior of the study participants. In addition, the authors hypothesized the role of gender and age differences in EC. They found empirical support for the hypothesis.

Next, Al Dilby and Farmanesh incorporated work-life balance and trust in leaders as the underlying mechanisms in the relationship between virtual leadership and job satisfaction in the post-COVID-19 era. The authors found that virtual leadership played a significant role in job satisfaction based on data collected from 196 IT employees across various industries, including finance, real estate, and trade data analysis companies. This effect was mediated by work-life balance and trust in the leader. Their finding throws light into the essential and positive role a leader plays, albeit virtually, in critical situations like COVID-19.

The paper by Sonnenschein et al. drew upon the self-determination theory and Herzberg's two-factor theory to understand how Norwegian and Danish newspapers represent employee motivation and job satisfaction of remote workers in light of the COVID-19 pandemic. The thematic analysis results established that the two countries' newspapers represented the topic of interest from different perspectives. The authors suggest that a hybrid model is an optimal solution for the future job market, where employees with task-based jobs can feel motivated and satisfied while working from home or the workplace.

In the study, Adekanmbi et al. invited nurses, midwives, doctors, and auxiliary services staff from the COVID-19 hospital ward, respiratory ICU, and outpatient clinics to examine how perceived organizational support, fear of COVID-19, and work-related stress affect safety performance. Interestingly, all the findings provide support for the hypotheses initially advanced. Based on these findings, the authors recommend that the policymakers in healthcare institutions ensure optimal work structure and schedule to help reduce workloads, which will go a long way in minimizing work-related stress and enhancing safety performance.

We are pleased that these six articles have been published in the Organizational Psychology section of the Frontiers in Psychology, and we believe that they have contributed significantly toward theory advancement. We hope this issue will inspire further research and discussion in industrial and organizational psychology and provide valuable insights to policymakers and practitioners alike. We wish like to thank the authors for their hard work and dedication. We also thank the reviewers and the editorial office for their invaluable feedback and guidance throughout the peer review process.

## Author contributions

All authors equally wrote and contributed to the manuscript. All authors contributed to the article and approved the submitted version.
